# Supplementary material for: Protein arginine methyltransferase 2 controls inflammatory signaling in acute myeloid leukemia
Source: Commun Biol. 2024 Jun 20;7:753. doi: 10.1038/s42003-024-06453-6 (PMC11190286; doi:10.1038/s42003-024-06453-6)
Supplement: Supplementary file 2 — Supplementary Information [file 42003_2024_6453_MOESM2_ESM.pdf]

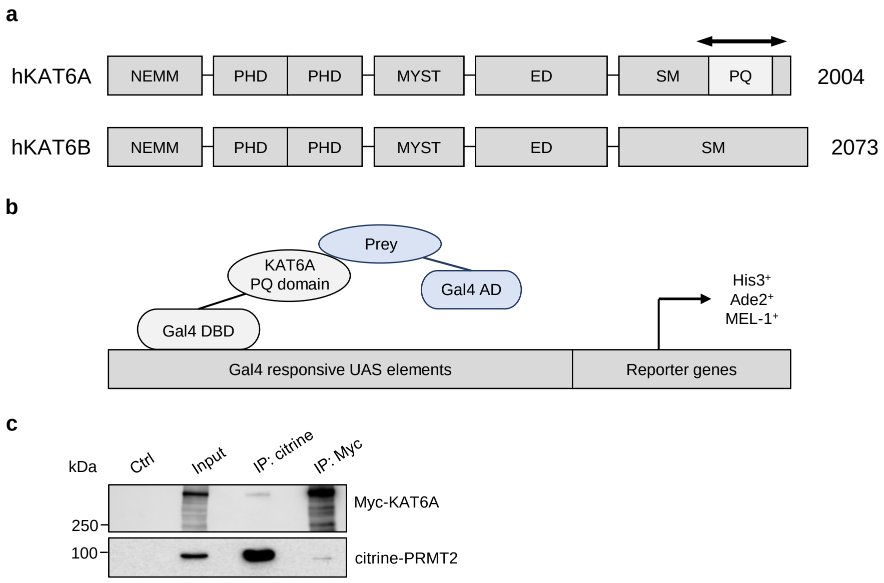

Supplementary Figure 1. (**a**) Schematic representation of human KAT6A and KAT6B proteins with amino acid lengths. (**b**) Schematic representation of the yeast two-hybrid system performed to identify PRMT2 as a partner of KAT6A. (**c**) Western Blot analysis after co-transfection followed by co-immunoprecipitation of either Myc-tagged KAT6A or citrine-tagged PRMT2 protein in HEK293 cells. The input fraction represents 5% of the total protein extract. The presented blot is representative of three independent experiments.

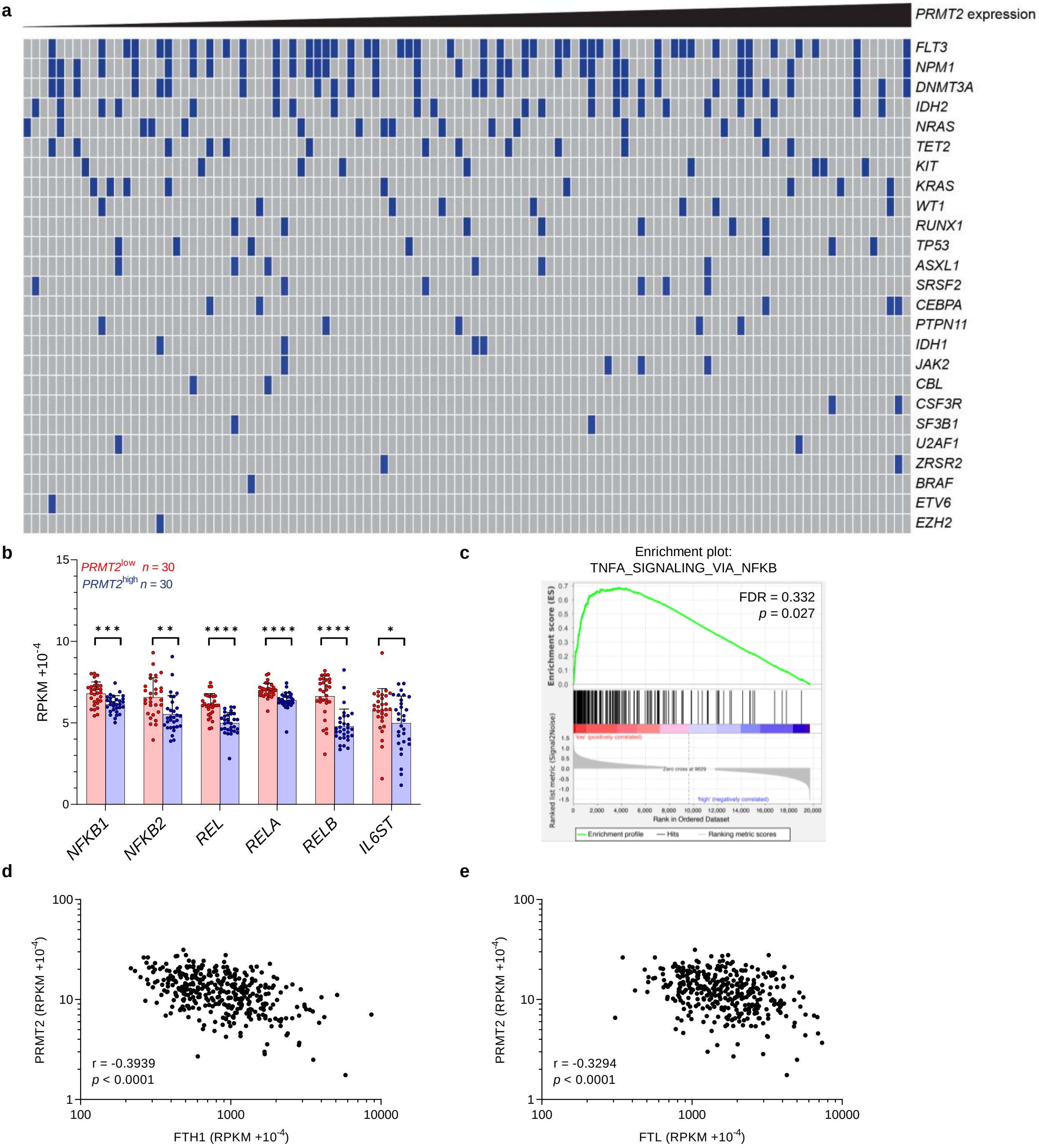

Supplementary Figure 2. (**a**) Mutation profile of the 105 AML patient exomes from *the Leucegene Project* depending on their *PRMT2* expression for a subset of frequently mutated genes in AML. Blue: mutated gene; grey: non mutated gene. (**b**) Box plots showing NF-κB-related gene expression of *PRMT2*^{low} and *PRMT2*^{high} patients in the *BeatAML* (n = 30 in each group) cohort. Error bars represent mean ± SD. Multiple unpaired t tests with p values indicated in the graphs; *p < 0.05; **p < 0.01; ****p < 0.0001 (**c**) GSEA plot for the "TNFA signaling via NFKB" hallmark on the *BeatAML* cohort with FDR and nominal p indicated. (**d**-**e**) Pearson's correlations of *PRMT2* expression with *FTH1* (d) and *FTL* expression (e) within *the Leucegene Project* cohort (n = 371).

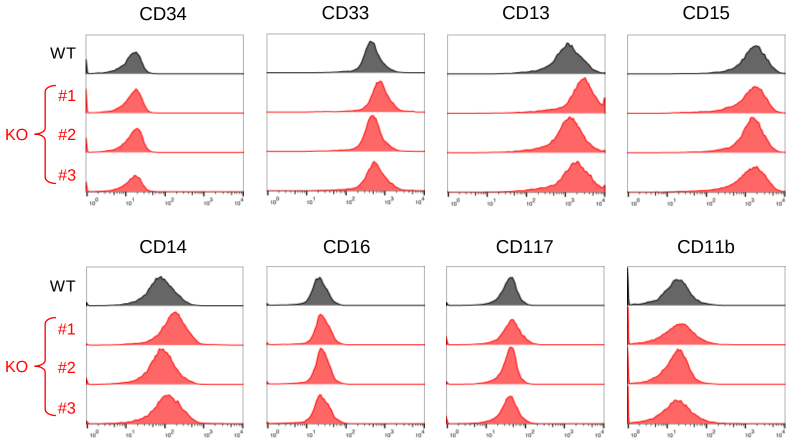

Supplementary Figure 3. Flow cytometry analysis showing fluorescence intensity for the CD34, CD33, CD13, CD15, CD14, CD16, CD117, and CD11b surface markers on WT HL-60 and 3 different clones of *PRMT2*^{KO} cells. Cells are considered as positive if the mean fluorescence intensity is above 10^2. The presented graphs are representative of three independent experiments.

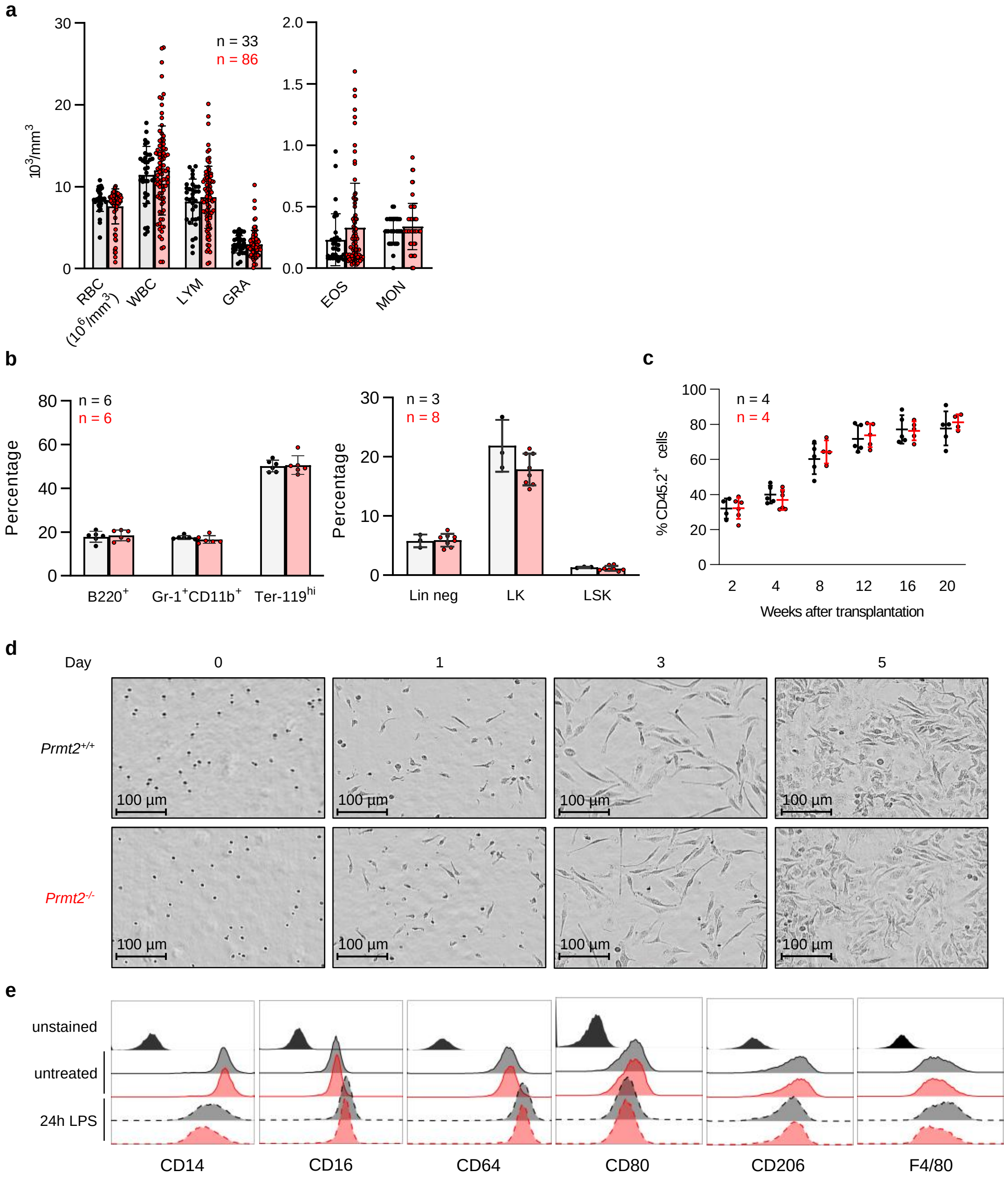

Supplementary Figure 4. (**a**) Blood cell counts in *Prmt2*$^{-/-}$ or control (*Prmt2*$^{+/+}$) mice. RBC: red blood cells; WBC: white blood cells; LYM: lymphocytes; GRA: granulocytes; EOS: eosinophiles; MON: monocytes. (**b**) Flow cytometry analysis on BM showing percentages of B cells (B220^{+}), monocytes/neutrophils (Gr-1^{+}CD11b^{+}), and erythroid cells (Ter-119^{hi}) (left) and lineage negative, LK and LSK (right). (**c**) Flow cytometry analysis of hematopoietic reconstitution of *Prmt2*$^{-/-}$ or control BM cells (CD45.2^{+}) transplanted into recipient mice (CD45.1^{+}) in competition with recipient's cells (1:1 ratio). Number of mice in each group are shown on graphs. All presented data (a-c) are expressed as mean ± SD. (**d**) Morphological analysis of monocytes during differentiation into macrophages. Images from phase-contrast microscopy using an Incucyte® S3 Live-Cell Analysis System at magnification x20. (**e**) Flow cytometry analysis for CD14, CD16, CD64, CD80, CD206, and F4/80 surface markers from unstimulated or 24h LPS-stimulated *Prmt2*$^{+/+}$ (grey) or *Prmt2*$^{-/-}$ BMDMs (red). Unstained cells are shown in black as negative control. Presented graphs are representative figures from three independent experiments.

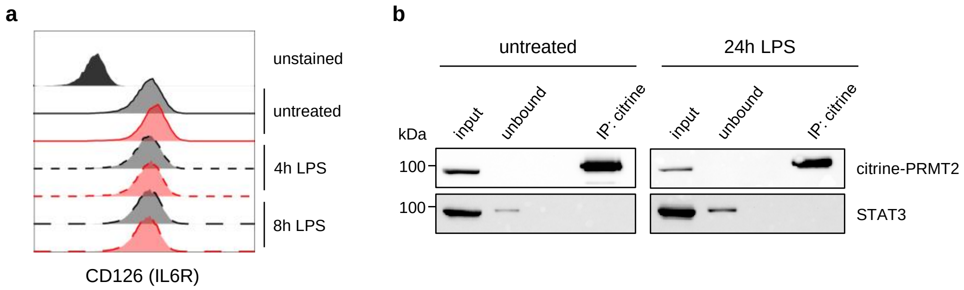

Supplementary Figure 5. (**a**) Flow cytometry analysis for the IL6 receptor (CD126) from unstimulated or LPS-stimulated WT (grey) or *PRMT2*^{KO} HL-60 cells (red). Unstained cells are shown in black as negative control. (**b**) Western Blot analysis of STAT3 after immunoprecipitation of citrine-PRMT2 unstimulated or 24h LPS-stimulated HL-60 cells. The input fraction represents 5% of the total protein extract. The presented blot is representative of three independent experiments.

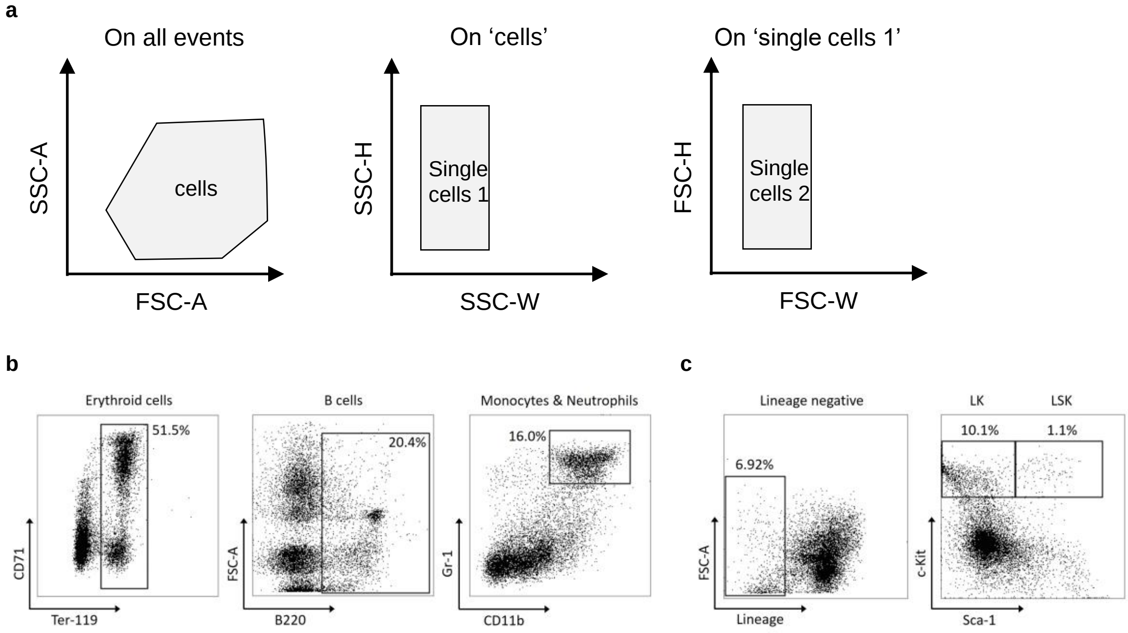

Supplementary Figure 6. (**a**) Gating strategy for all the presented flow cytometry data, from all events. (**b-c**) Gating strategies for Supplementary Figure 4b, from the 'single cells 2' gate.

Full unedited blot for Supplemental Figure 1c

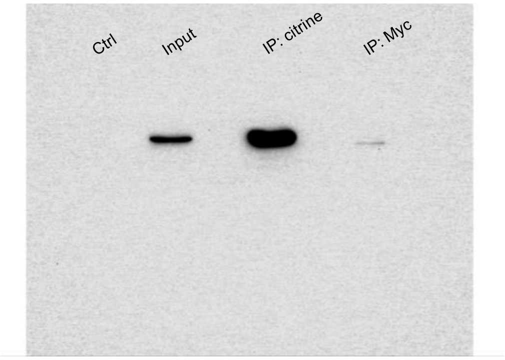

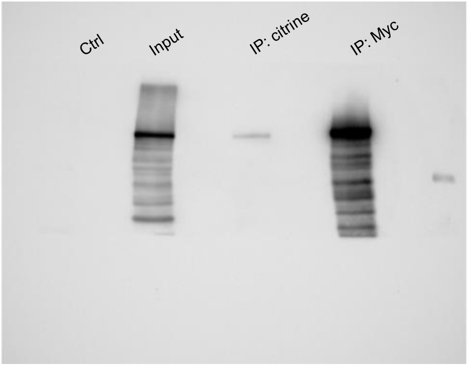

Anti-GFP

Anti-MYC

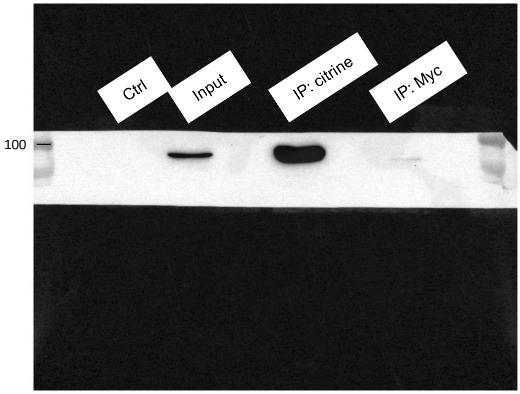

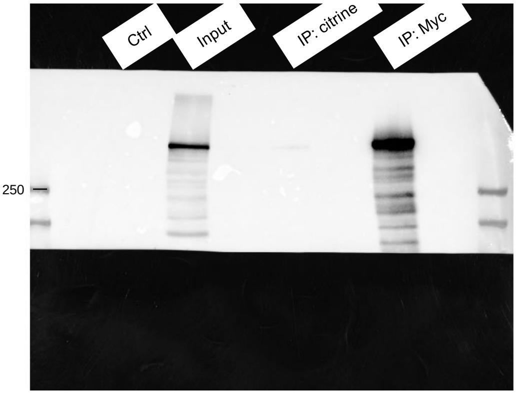

Supplementary Figure 7. All uncropped Western Blot images displayed in the manuscript.

Full unedited blot for Figure 3a

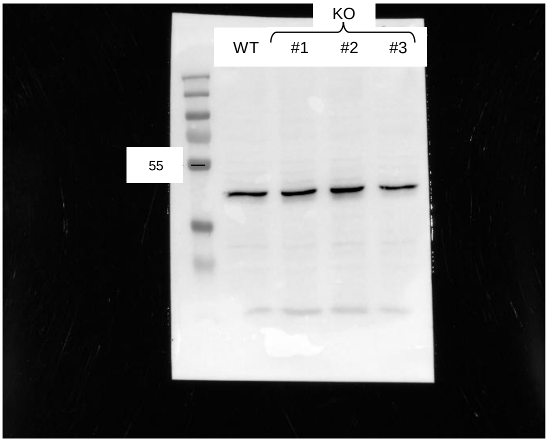

Anti-PRMT1

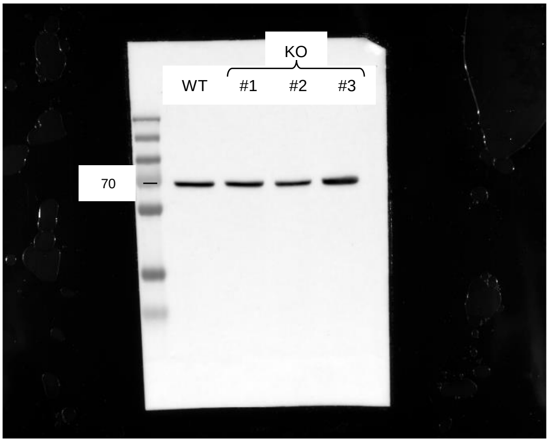

Anti-PRMT3

Supplementary Figure 7. All uncropped Western Blot images displayed in the manuscript.

Full unedited blot for Figure 3a

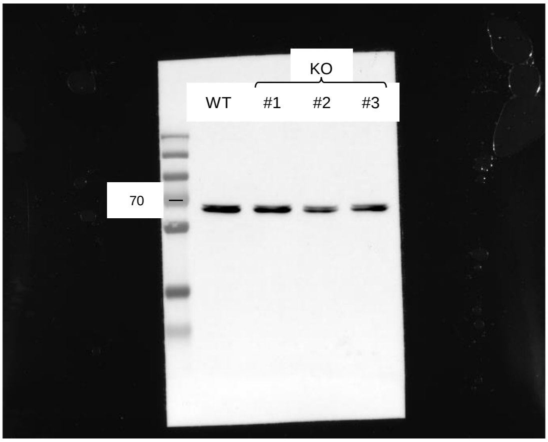

Anti-CARM1

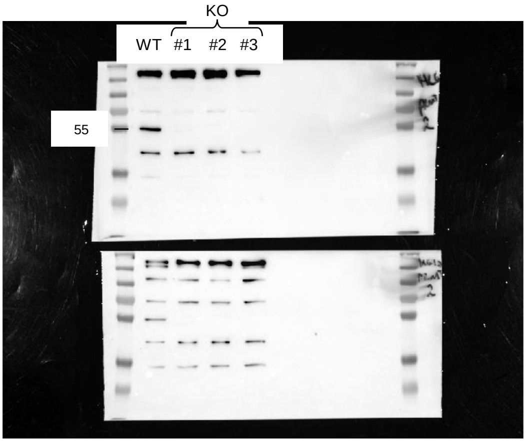

Anti-PRMT2

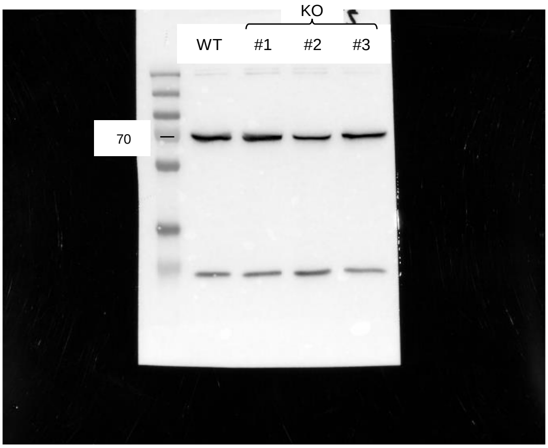

Anti-PRMT5

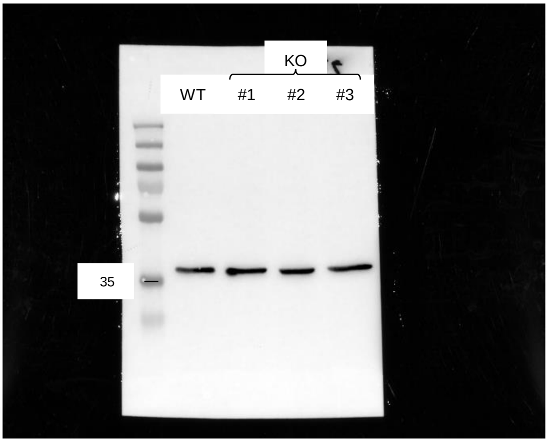

Anti-GAPDH

Supplementary Figure 7. All uncropped Western Blot images displayed in the manuscript.

Full unedited blot for Figure 5c

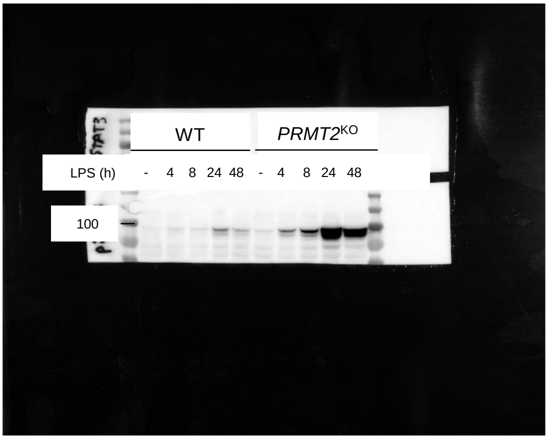

Anti-pSTAT3

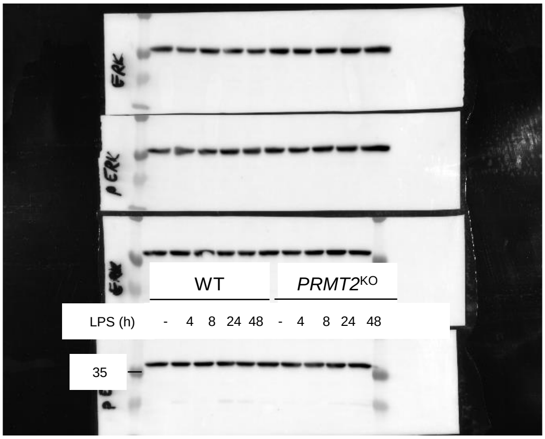

Anti-GAPDH

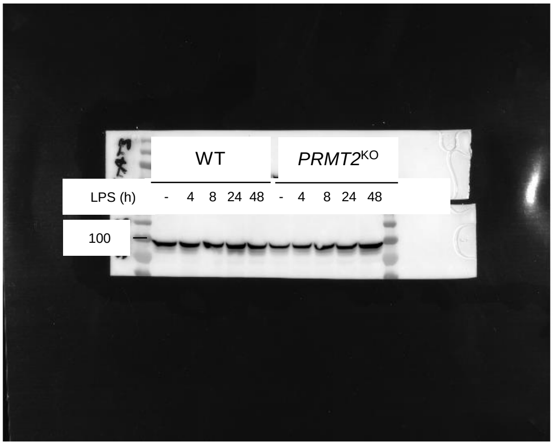

Anti-STAT3

Supplementary Figure 7. All uncropped Western Blot images displayed in the manuscript.

Full unedited blot for Figure 5e

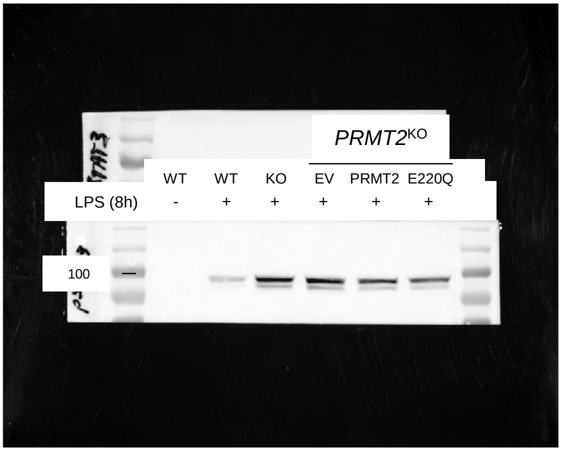

Anti-pSTAT3

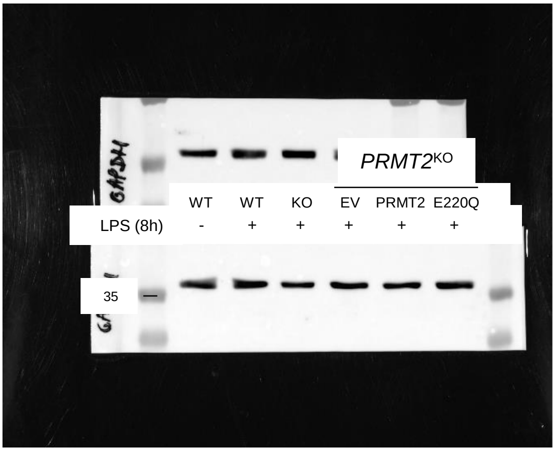

Anti-GAPDH

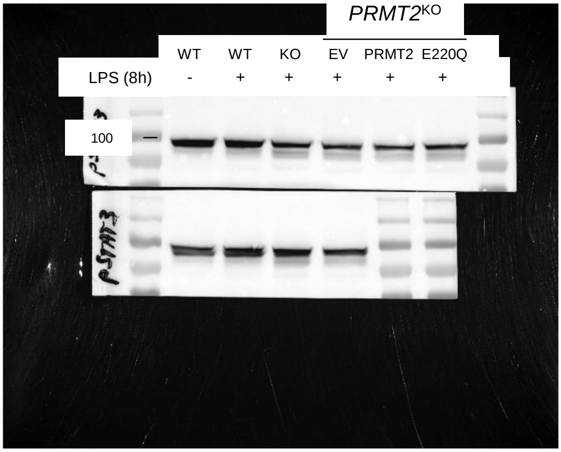

Anti-STAT3

Supplementary Figure 7. All uncropped Western Blot images displayed in the manuscript.

Full unedited blot for Figure 6a

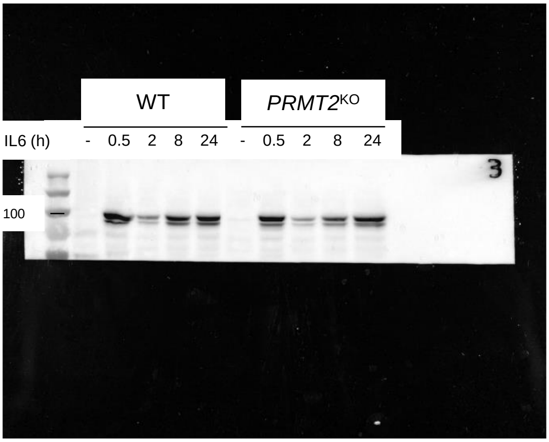

Anti-pSTAT3

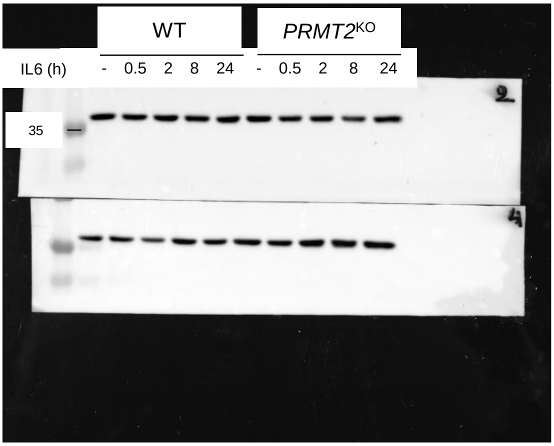

Anti-GAPDH

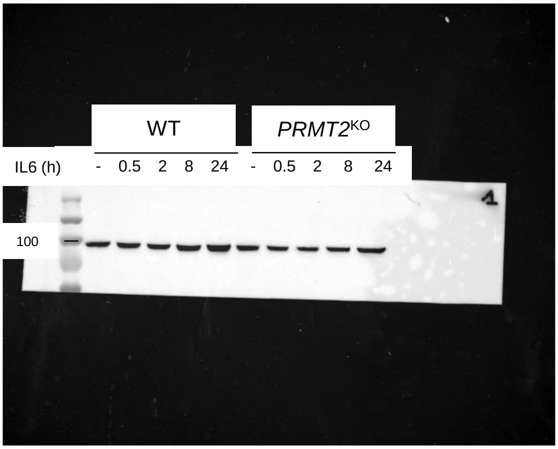

Anti-STAT3

Supplementary Figure 7. All uncropped Western Blot images displayed in the manuscript.

Full unedited blot for Figure 6c

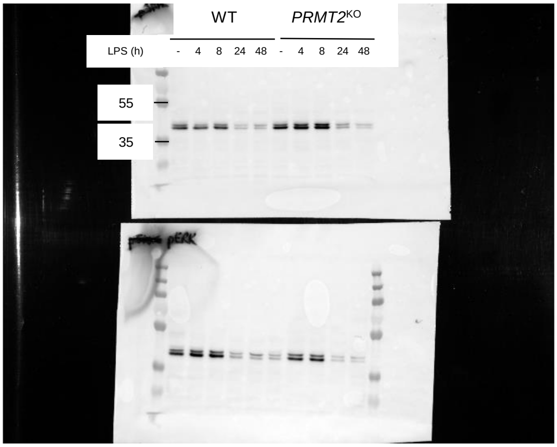

Anti-p-ERK1/2

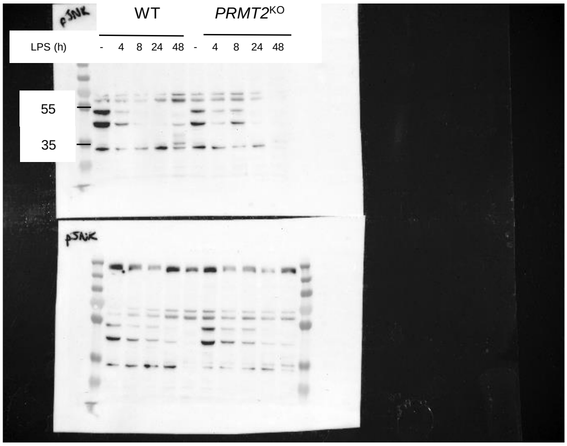

Anti-p-SAPK/JNK

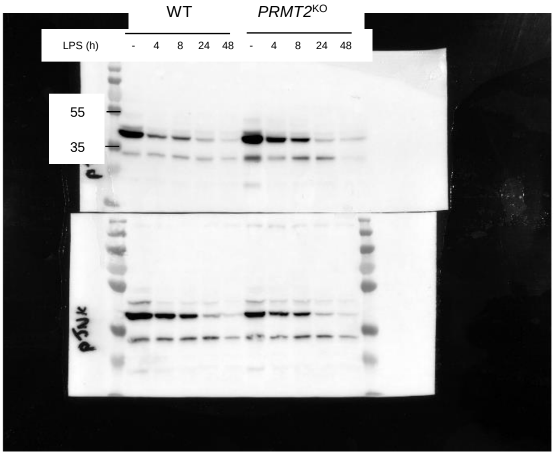

Anti-p-P38

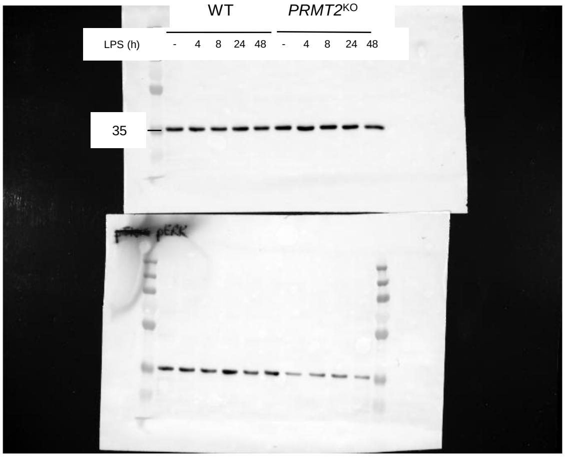

Anti-GAPDH

Supplementary Figure 7. All uncropped Western Blot images displayed in the manuscript.

Full unedited blot for Figure 6c

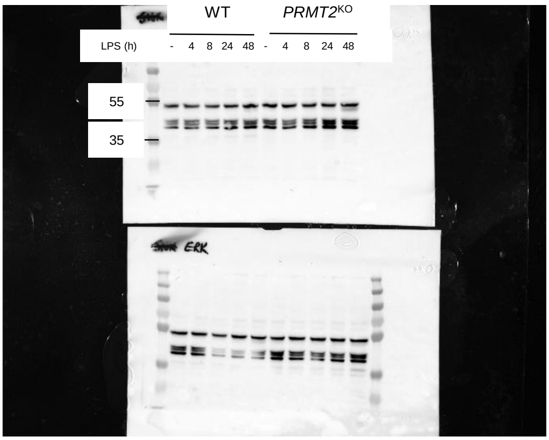

Anti-ERK1/2

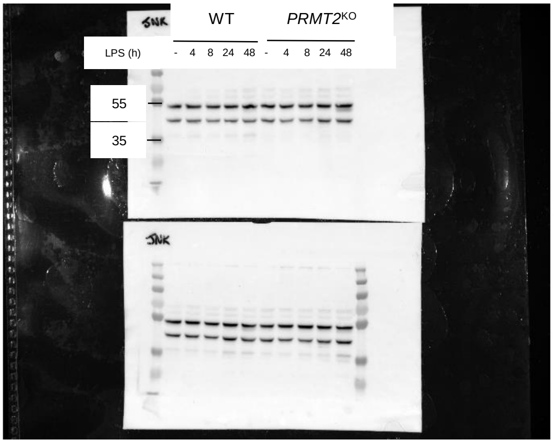

Anti-SAPK/JNK

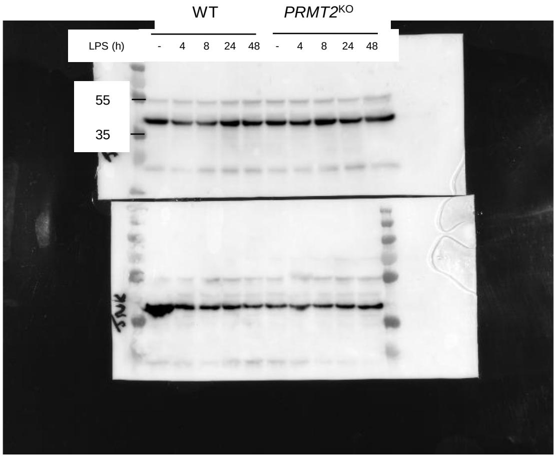

Anti-P38

Supplementary Figure 7. All uncropped Western Blot images displayed in the manuscript.

Full unedited blot for Figure 6d

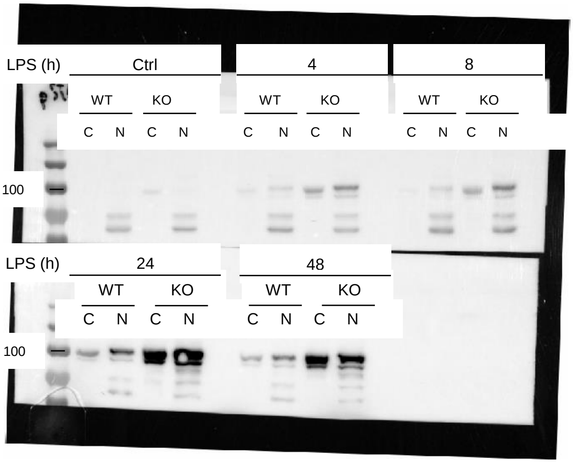

Anti-pSTAT3

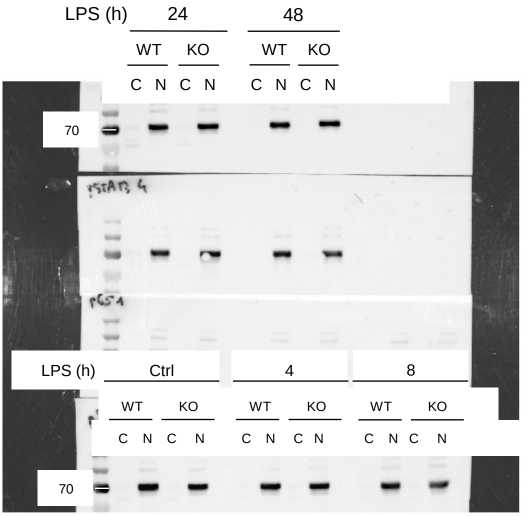

Anti-SP1

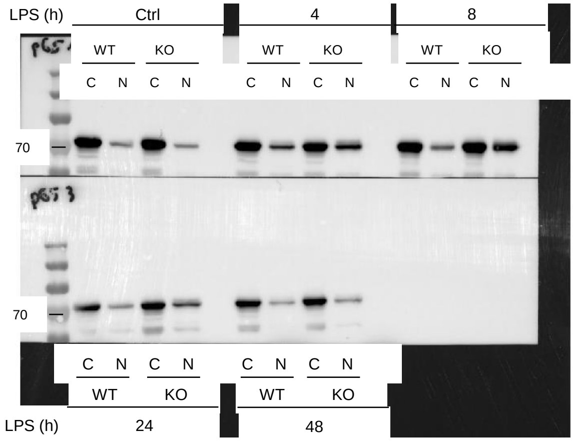

Anti-NFKB P65

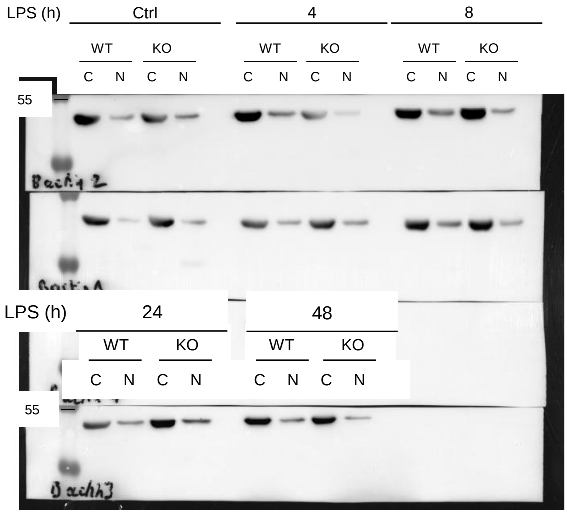

Anti-ACTB

Supplementary Figure 7. All uncropped Western Blot images displayed in the manuscript.

Full unedited blot for Supplemental Figure 5b

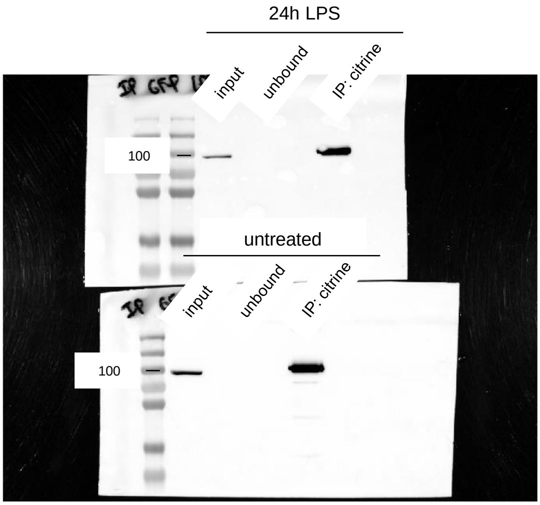

Anti-GFP

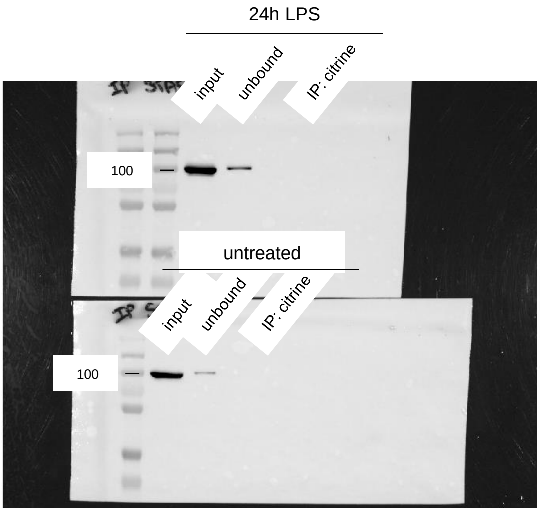

Anti-STAT3

Supplementary Figure 7. All uncropped Western Blot images displayed in the manuscript.
