# Supplementary material for: Protein arginine methyltransferase 2 controls inflammatory signaling in acute myeloid leukemia
Source: Commun Biol. 2024 Jun 20;7:753. doi: 10.1038/s42003-024-06453-6 (PMC11190286; doi:10.1038/s42003-024-06453-6)
Supplement: Supplementary file 3 — Description of additional supplementary files [file 42003_2024_6453_MOESM3_ESM.pdf]

Description of Additional Supplementary Files

File name: Supplementary data 1

Description: The source data behind the graphs in the paper
